# Proof-of-concept for a novel nanotechnology-based treatment for urolithiasis

**DOI:** 10.1007/s00240-024-01564-5

**Published:** 2024-04-06

**Authors:** Ian Houlihan, Benjamin Kang, Vijay Krishna, Smita De

**Affiliations:** 1 Biomedical Engineering Department, Lerner Research Institute, Cleveland Clinic, Cleveland, OH 44195 USA; 2 Biomedical Engineering Department, Cleveland Clinic Lerner College of Medicine, Case Western Reserve University, Cleveland, OH 44106 USA; 3 Urology Department, Glickman Urology and Kidney Institute, Cleveland Clinic, Cleveland, OH 44195 USA; 4 Urology Department, Cleveland Clinic Lerner College of Medicine, Case Western Reserve University, Cleveland, OH 44106 USA

**Keywords:** Kidney, Nanotechnology, Urolithiasis, Laser lithotripsy

## Abstract

Proof-of-concept of photonic lithotripsy in an in vitro setting and its ability to fragment the most common stone types is demonstrated. Effectiveness of different classes of photonic nanoparticles in fragmenting human stones is assessed. De-identified human stones were collected after institutional approval. Stones of a size range between 2–4 mm were rehydrated in simulated urine for 24 h. Stones were then coated with a solution of nanoparticles prior to activation with either a 785 nm or 1320 nm near-infrared energy source. Photonic lithotripsy achieved greater than 70% success rate in fragmentating calcium oxalate monohydrate stones using carbon-based nanoparticles for both near-infrared wavelengths. For gold-based nanoparticles, there was a similar success rate with the 785 nm wavelength but a significant decrease when using the 1320 nm wavelength energy source. All stones fragmented with the energy source at a distance ≥ 20 mm from the stone’s surface. Limitations include the use of mixed-composition stones, a lack of complete stone immersion in liquid during treatment, and smaller stone size. Different classes of nanoparticles when excited with a near-infrared energy source can fragment common stone types in vitro. This technology has the potential to change the way we approach and treat patients with urolithiasis in a clinical setting.

## Introduction

The treatment of urolithiasis changed significantly from morbid open operations to minimally and non-invasive procedures with the development of shockwave lithotripsy and endoscopic techniques in the 1980’s [1]. However, since the proliferation of laser lithotripsy via ureteroscopy, currently the most common stone procedure in the US, there has been little to no shift away from the use of lasers that are highly absorbed by water and need to be extremely close to a stone’s surface (≤ 2mm) for effective fragmentation [2–4]. This trend has continued with the introduction of the thulium fiber laser (TFL), which has a higher absorption by water than the well-established holmium:yttrium–aluminum–garnet (Ho:YAG) laser [5]. While effective, there are a number of downsides to these lasers, including potential for tissue damage due to thermal effects, stone retropulsion, need for direct stone contact, and often limited ability to treat stones in certain anatomic locations such as lower pole stones [6–8]. We propose a novel technique, termed photonic lithotripsy, for treating kidney stones using nanotechnology.

Nanotechnology refers to the manipulation of materials on a scale of 10^–9^ or one billionth of a meter. Materials at this size exhibit unique properties that are not seen from the same material when present at a larger volume [9]. For example gold particles on a nanoscale can act as a catalyst in a chemical reaction to facilitate oxidation and reduction reactions [10]. This is not seen with gold as a bulk material. Carbon and gold-based nanoparticles are becoming powerful tools for use in the medical field, with applications ranging from cancer treatments to enhanced imaging capabilities [11–13]. The first use of gold nanoparticles as a treatment for prostate cancer is currently in clinical trials [14]. The nanoparticles used in this study have unique properties in that when excited by near-infrared (NIR) energy, they generate vibrations. These vibrations result in heat and acoustic or mechanical energy. These unique properties led us to hypothesize that the vibrational energy (thermal and acoustic) produced by these nanoparticles when excited with low intensity NIR energy can fragment common types of human kidney stones in an in vitro setting.

## Materials and methods

### Concept

**Fig. 1:**
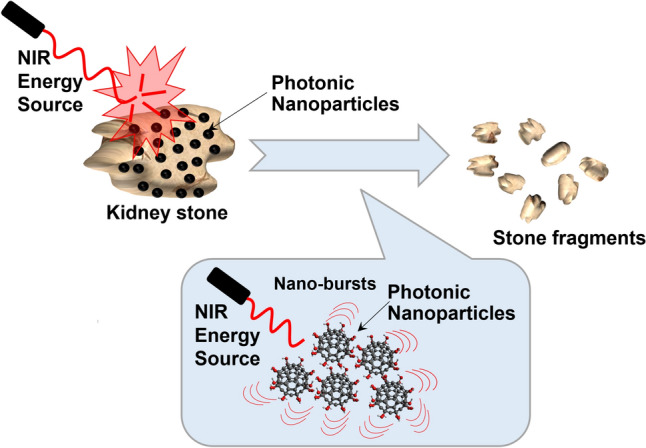
Schematic of the fundamental mechanism of this technology. The stones are coated in a nanoparticle solution and exposed to a NIR energy source which produces nano-bursts in the form of thermal and acoustic energy that ultimately lead to the failure of the stone

The proposed mechanism for photonic lithotripsy is shown in Fig. 1. First, the kidney stone is coated with a nanoparticle solution. These nanoparticles are then excited with a NIR energy source supplied in the form of a coherent and continuous waveform (i.e., a laser) that is minimally absorbed by water, thus decreasing risk of thermal injury. The lasers used for exciting the photonic nanoparticles in this study had wavelengths of 785 nm or 1320 nm and were delivered through a 550-micron optic fiber. This laser system could be adapted for clinical use with fibers similar to current practice. These wavelengths were chosen because 785 nm and 1320 nm are minimally absorbed by water, about 100–1000 times less than lasers currently used for lithotripsy [15]. Once excited, the nanoparticles vibrate, generating heat and acoustic energy that results in the breakdown of the stone. In contrast to current clinical lasers, the energy delivered by the lasers alone is not sufficient to cause stone fragmentation.

### Tested nanoparticles

**Fig. 2:**
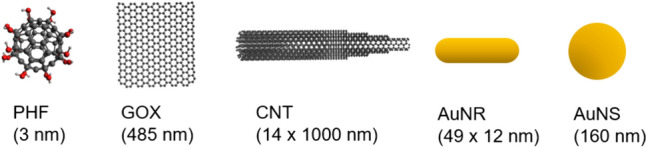
Schematic of each nanoparticle used in this study with their approximate size. Three carbon-based (PHF, GOX and CNT) and two gold-based (AuNR and AuNS) nanoparticles were used

There were five different nanoparticles used in this study: three carbon-based and two gold-based. Figure 2 shows a schematic of these nanoparticles with their corresponding size. The specific nanoparticles tested were polyhydroxy fullerenes (PHF), graphene oxide (GOX), carboxylated multi-walled carbon nanotubes (CNT), gold nanorods (AuNR), and gold nanospheres (AuNS). These nanoparticles were chosen based on their known ability to convert light energy to heat and/or acoustic energy when excited by NIR energy [11, 16–20].

### In vitro stone fragmentation

**Fig. 3:**
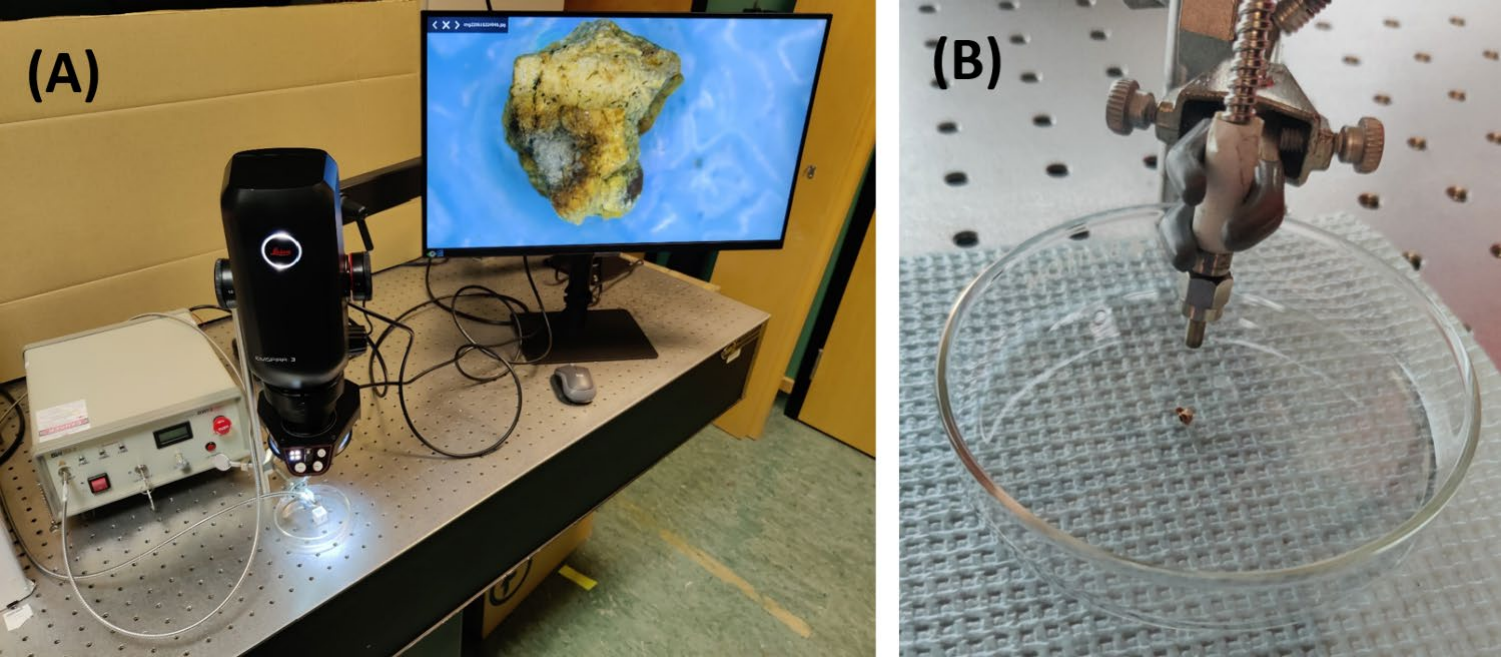
(**A**) Experimental set up for in vitro testing of photonic lithotripsy included the NIR energy source (on left), stereomicroscope with the petri dish below it for holding the stones (center) and screen for stone imaging (right). (**B**) Zoomed in image of the petri dish and laser fiber delivery

De-identified human kidney stones were collected from the pathology laboratory at our institution after receiving internal review board approval. The composition of each stone was determined by the pathology laboratory using Fourier transform infrared (FTIR) spectroscopy. Stones were sorted by sieving using different sizes of mesh ranging between 2–4 mm. The in vitro set up used for these experiments is shown in Fig. 3. First, the human stones were rehydrated in simulated urine for 24 h prior to testing, after which the stones were placed in a petri dish and 10 μl of the test nanoparticle solution was applied to the stone (simulated urine was used for the control samples). The concentrations of the nanoparticle solutions were chosen to ensure that the same amount of light absorption was achieved by each solution at a given wavelength, where PHF at 10 mg/mL was the baseline (see Table 1). The absorbance was calculated by measuring the amount of light absorbed by the different nanoparticle solutions at the given wavelengths using a laser power detector (Coherent, Santa Clara, CA).

**Table 1:** Concentration and corresponding absorbance value for each nanoparticle solution at 785 nm and 1320 nm wavelengths

Nanoparticle	785 nm		1320 nm	
	Concentration (mg/mL)	Absorbance (AU)	Concentration (mg/mL)	Absorbance (AU)
PHF	10	1.24	10	0.84
GOX	0.25	1.33	0.015	0.82
CNT	0.03	1.04	0.015	0.91
AuNR	0.015	1.37	0.04	0.83
AuNS	0.06	1.33	0.015	0.84

The nanoparticles were excited with a 785 nm (B&W TEK Inc., Newark, DE) or 1320 nm (QPC Lasers, Sylmar, CA) NIR laser energy at a distance of at least 20 mm. The stones were irradiated with the specified NIR energy for a total of 9 min, in 3-min intervals. The power settings used were 2 W and 4 W for the 785 nm and 1320 nm wavelength sources, respectively. The stones were continuously imaged using an Emspira 3 Leica stereomicroscope. Controls were also performed in which stones were exposed to the NIR energy coated in 10 μl of simulated urine.

### Micro-computed tomography (CT)

To assess stone failure and crack formation, the stones were scanned in a GE eXplore Locus RS micro-CT pre- and post-irradiation. Settings for the micro-CT scans were 80 kVp, 490 mA with a voxel size of 20 µm. Data obtained from the micro-CT scans were exported and analyzed using Slicer software [21]. The surface area and Hounsfield unit were calculated based on a segmentation volume which only included the stone.

## Results

**Fig. 4:**
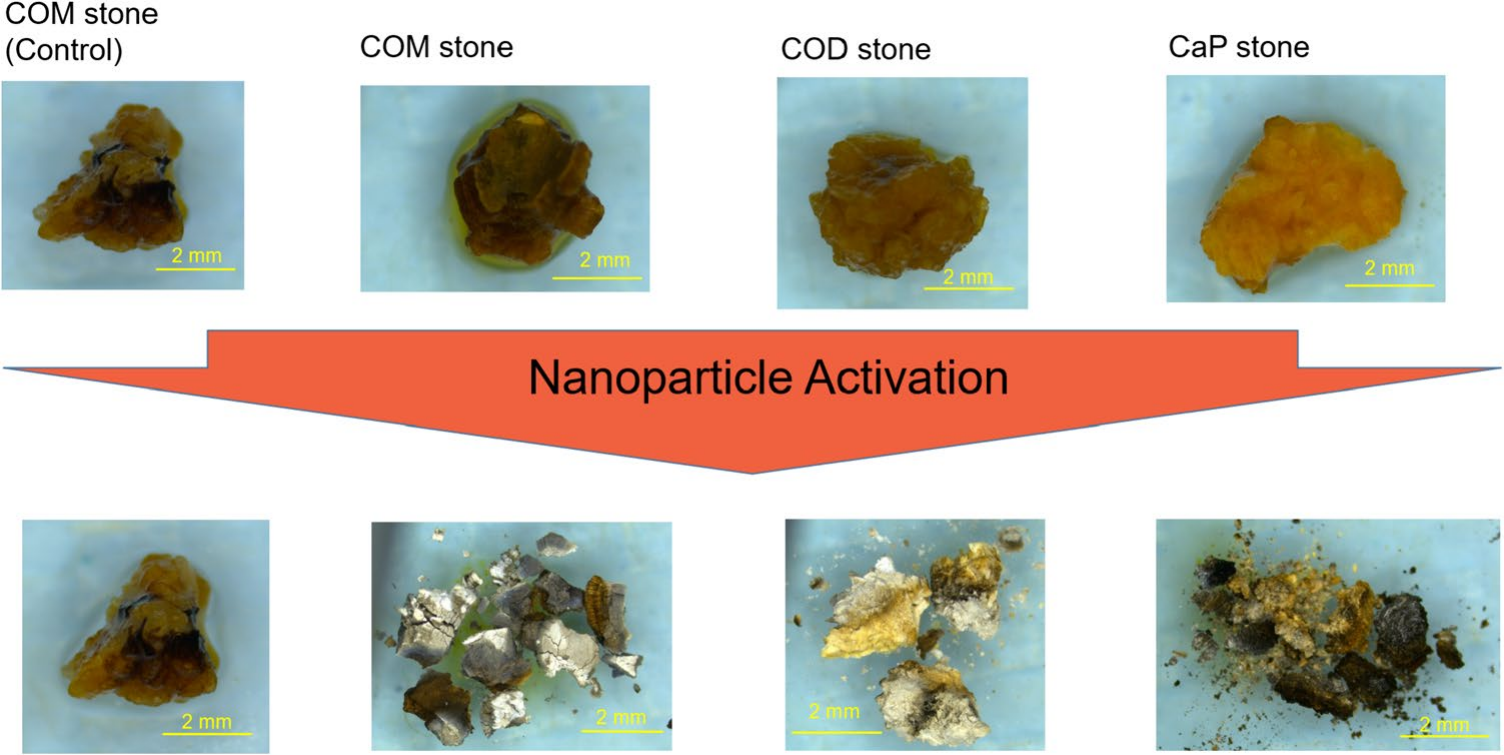
Proof-of-concept images showing three of the major stone types (COM, COD, CaP) treated with PHF at a concentration of 10 mg/mL and a control exposed to the 785 nm wavelength energy source at a power of 2 W. Images show pre and post treatment of the same stone, each stone was exposed for under 3 min. The brown pigmentation is due to the color of the PHF solution

In this proof-of-concept study evaluating the potential use of nanotechnology for fragmentation of kidney stones, we were able to successfully demonstrate in vitro fragmentation of the most common stone types (i.e., calcium oxalate monohydrate (COM), calcium oxalate dihydrate (COD), and calcium phosphate (CaP) stones). Figure 4 illustrates examples of stones that were treated with the 785 nm energy source and a PHF solution of 10 mg/mL. Considerable fragmentation and dusting of the stones (4 mm pre-treatment) is seen with no fragments > 2 mm remaining. Control experiments showed no fragmentation or breakdown of stones (further discussed below).

**Fig. 5:**
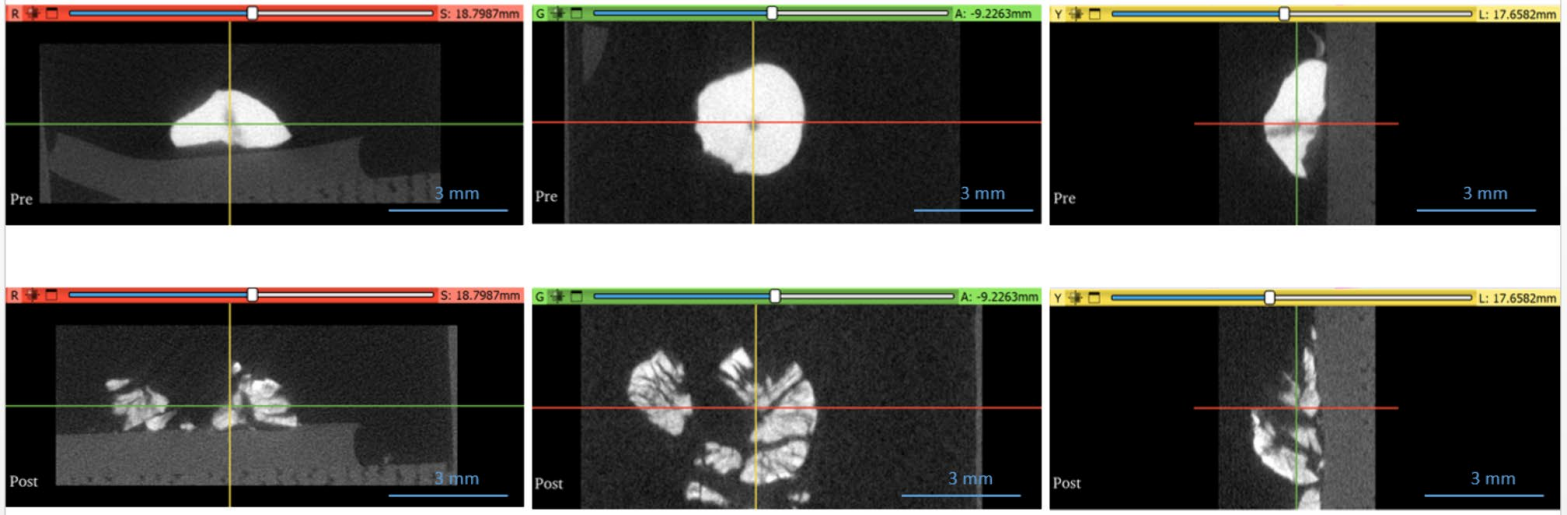
Micro-CT images of a COM stone pre (top images) and post (bottom images) exposure to a 1320 nm energy source at 4 W after being coated with PHF at a concentration of 10 mg/mL. Crosshairs shown on the images represent the slice from the corresponding color view i.e., the red-crosshair line represents the image slice shown in the red-image pane

The degree of fragmentation was further examined using micro-CT. The micro-CT images for a COM stone treated with a PHF nanoparticle solution and the 1320 nm NIR energy source is shown in Fig. 5. As with the previous images of the stones shown in Fig. 4, there is significant breakdown of the stone after treatment with additional cracks throughout suggesting further stone failure.

**Fig. 6:**
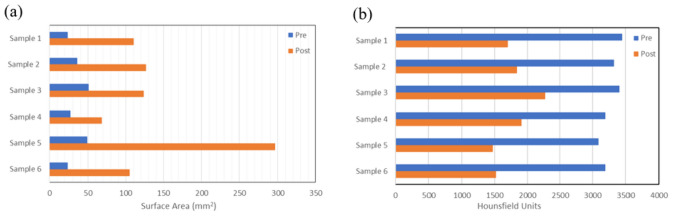
(**a**) Surface area of the six different COM stone samples coated with PHF pre-(blue) and post-(orange) exposure to the 1320 nm energy source (**b**) Hounsfield units of the six COM stone samples pre-(blue) and post-(orange) treatment

This breakdown of the stones is also demonstrated in the analysis of the micro-CT data in Slicer software. The plots in Fig. 6 demonstrate the change in surface area pre- and post-irradiation for six different stones tested. There is a noticeable increase in the total stone surface area and a reduction in the mean Hounsfield units on the micro-CT images after treatment reflecting the fragmentation and dusting of the stones.

**Fig. 7:**
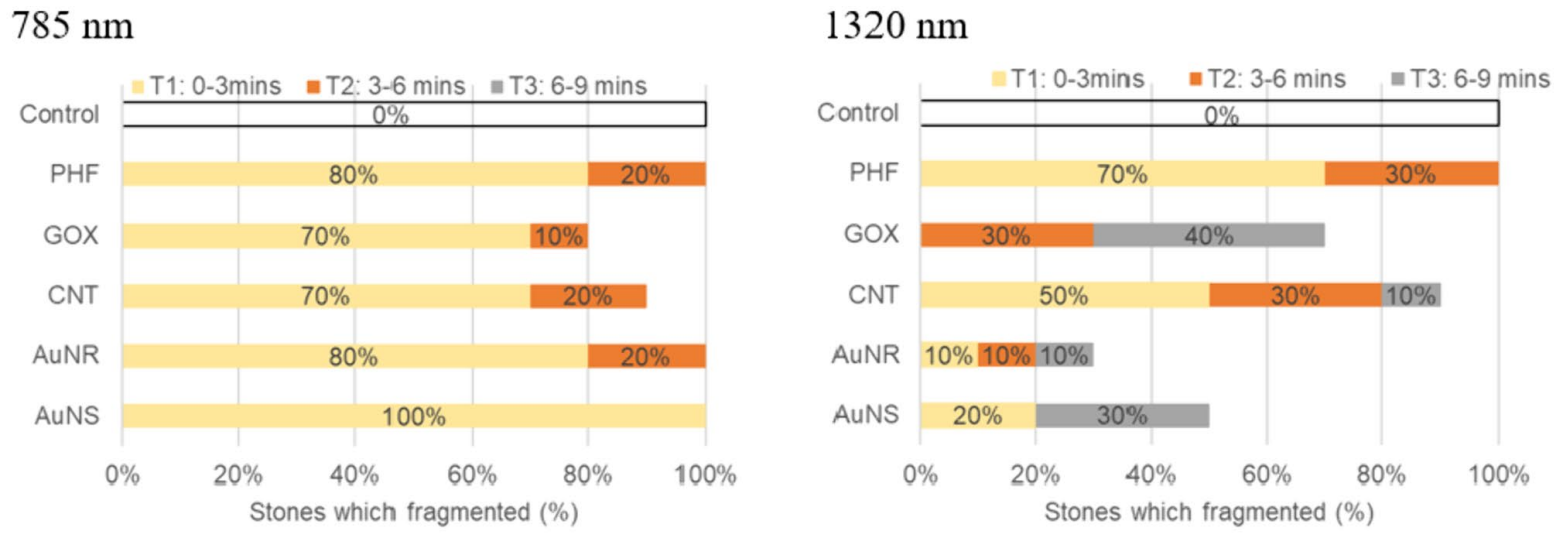
Comparison of the time period in which fragmentation of the COM stones occurred using a 785 nm (left) or 1320 nm (right) energy source with different photonic nanoparticles. Exposure times were broken into three periods over a total of 9 min, where *n*  =  10 stones was used for each nanoparticle tested, this data has been reported previously [22]

Several different nanoparticles were tested to investigate if there were any differences in their effectiveness for fragmenting stones. Figure 7 shows the percentage of stones that fragmented for each nanoparticle and NIR wavelength in a given time period (T1: 0–3 min, T2: 3–6 min and T3: 6–9 min).

## Discussion

We propose a novel technique that uses a non-contact, low intensity, continuous wave energy source with minimal absorption by water to excite nanoparticles that generate thermal and acoustic energy on the surface of stones resulting in stone failure. In this proof-of-concept study, we have demonstrated for the first time the ability to successfully fragment common types of kidney stones using the described photonic lithotripsy. The NIR energy source used in this study is not directly responsible for the fragmentation of the stones but is used to excite the nanoparticles as confirmed by our control specimens.

Notably, the tested nanoparticles can fragment stones with the NIR energy being up to 20 mm from the stone surface (can be shorter), and at power settings of less than 5 W (compared to clinical lasers which use power settings up to 100 W) [6]. These findings are exciting in considering potential future applications.

To better understand how this technology fragments kidney stones, it was important to analyze the same stone pre- and post-treatment. This was successfully achieved using observational data and micro-CT analysis. The micro-CTs showed extensive cracking throughout the stone samples after treatment, which corresponded to an increase in surface area post-irradiation due to new surface creation. There was also a decrease in the Hounsfield units, for which we propose two different mechanisms. One is the breakdown and ultimate reduction in density as the stone fragments, and the other is a change in the mineral composition of the stone due to the thermal energy generated by the nanoparticles (this thermal degradation has been shown previously using FTIR [22]). Use of micro-CT in identifying mineral composition of stones has been reported in the literature, although it has not been used to determine mineral compositional changes pre and post stone treatment [23, 24]. Both compositional change and stone fragmentation can have a significant effect on HU and therefore, it is challenging to differentiate between the two mechanisms in a quantitative manner. The proposed mechanism of stone failure and the contribution of these effects has been outlined in more detail previously using scanning electron microscopy and thermal imaging during photonic lithotripsy [22]. Based on these studies, current byproducts of this technology appear to be predominantly thermal in nature, although these nanoparticles can be further engineered to enhance the acoustic or mechanical effect for fragmentation (and potentially decrease the thermal effect) as well as modified with ligands to directly target stones [11, 12].

The specific NIR energy source used had a noticeable effect on the effectiveness of photonic lithotripsy as did the duration of irradiation and type of nanoparticle used (Fig. 7). For example, there was a lower fragmentation rate with the gold nanoparticles when irradiated by the 1320 nm energy source. This may be attributed to the NIR wavelength used since these gold nanoparticles were designed to have peak absorption at 808 nm and therefore absorb significantly lower amounts of energy outside of this region.

It is encouraging to note that our proof-of-concept study on photonic lithotripsy demonstrated a 70% or greater fragmentation rate across both NIR energy sources, with the exception of the gold nanoparticles and the 1320 nm wavelength, as discussed above. The 785 nm energy source appears to perform the best across all the nanoparticles tested with the majority of stones fragmenting in the first 3-min time period. In the control groups (stones rehydrated with simulated urine but not treated with nanoparticles), none of the 20 stones tested across both wavelengths resulted in fragmentation, highlighting that the NIR energy sources excite the nanoparticles and do not contribute to the fragmentation of the stones.

We envision initial trials with photonic lithotripsy to be performed similar to current clinical practice with patients under anesthesia and NIR energy delivered via a ureteroscope as there is a lack of data regarding potential pain during photonic lithotripsy. However, the wavelengths used in this study are minimally absorbed by water, unlike the current clinical lasers, which gives these NIR energy sources the ability to penetrate tissue with minimal adverse effects, suggesting the potential for this technology to be used extracorporeally. For example, nanoparticles could be delivered to the site of the stone and excited extracorporeally with a low intensity energy source that can penetrate tissue. Extracorporeal use with the current wavelengths would theoretically be for stones up to 3.5 cm from the skin surface, which would only be applicable to a limited number of circumstances [11, 12, 25, 26]. Future development of this technology would incorporate energy sources with deeper penetration capabilities, such as radio frequency energy (20–200 cm depending on the frequency used) [27].

The nanoparticles used in this study are electrostatically bound to the stones, but a stronger interaction between the nanoparticles and the stone’s surface would result in a more efficient transfer of energy from the nanoparticles to the stone, and theoretically, a quicker fragmentation rate. This could also reduce the risk of tissue damage through improved binding to the stone compared to the surrounding tissue. This concept has been proposed previously by Ramaswamy et al. with microbubbles functionalized with bisphosphonates to more effectively bind to kidney stone surfaces [28]. We are currently designing nanoparticles with ligands that will allow for such ‘targeting’ of stones.

Similar to this work and the microbubble-based technology referenced above, others also aim to disrupt the current standards for the treatment of urolithiasis. Perhaps the most developed at this time is Burst Wave lithotripsy (©Sonomotion, San Mateo, CA). In Burst Wave lithotripsy, stones are identified via ultrasound, and then fragmented using low pressure ultrasound waves [29]. Currently, this technology is being used in clinical trials as an investigational device. While results have been promising, a key limitation is the need to use ultrasound for pinpoint localization of stones as identification of stones via ultrasound is challenging [30, 31]. In photonic lithotripsy, we anticipate being able to use various modalities for intra-procedural imaging including fluoroscopy, ultrasound, and direct visualization.

While this study has successfully demonstrated the ability of nanoparticles to fragment human kidney stones using low energy, non-contact energy sources, several limitations must be noted. First, the tested stones were obtained during surgical procedures for stone treatment so there is a potential for stone alteration due to the lithotripsy technique used intraoperatively. However, we decided not to use simulated stones (such as Bego stones, BEGO USA, Inc.) to assess this technology given the ultimate goal to target stones and simulated stones are predominantly designed to match the mechanical and not chemical properties of a human kidney stone. Second, some of the stones used were of mixed composition, although the use of pure stones would limit the amount of stones available for testing as less than 10% of stones are reportedly composed of a single component [32]. Third, stones were tested in an in vitro environment. Future studies are planned to evaluate photonic lithotripsy with stones immersed in simulated urine. Fourth, the time periods chosen for energy exposure were not refined and additional optimization of timing as well as quantification of post-treatment fragment sizes will allow for more precise assessment of the different variables involved. Finally, only small-sized stones were tested for proof-of-concept and so evaluation of larger stones would be needed to translate this technology into a clinical setting.

Future directions include addressing several of the above limitations and preparing for in vivo testing. Safety studies in urothelial cell cultures as well as small animal models will be needed. We do not anticipate there to be toxicity concerns as prior in vivo studies have demonstrated the safety of these nanoparticles, including for oncologic applications [11, 33–35]. Various parameters will need to be optimized such as nanoparticle concentrations. In addition, nanoparticles that can specifically bind to stones may improve efficiency and decrease potential for tissue damage, as previously discussed. Ultimately, our goal is to assess the efficacy of photonic lithotripsy using the targeting nanoparticles in a porcine model and compare with current standard of care.

## Conclusion

Carbon- and gold-based nanoparticles excited by a non-contact, low-power NIR energy source can fragment common types of human kidney stones in vitro. This proof-of-concept study suggests the potential for using photonic lithotripsy as a novel approach for treating kidney stones.

## Data Availability

The data is available upon request.
